# Meta-analysis reveals pathological complete response benefits from neoadjuvant immuno-chemotherapy combination in patients with HER2-negative breast cancer

**DOI:** 10.1186/s12885-026-16230-9

**Published:** 2026-06-08

**Authors:** Yuhan Wei, Yalong Qi, Hewei Ge, Cheng Zeng, Qin Li, Fei Ma

**Affiliations:** 1 Department of Medical Oncology, National Cancer Center/National Clinical Research Center for Cancer/Cancer Hospital, Chinese Academy of Medical Sciences and Peking Union Medical College, No. 17 Panjiayuan Nanli, Beijing, 100021 China; 2 Department of Oncology, Beijing Friendship Hospital, Capital Medical University, Beijing, 100050 China

**Keywords:** Breast cancer, Neoadjuvant immunotherapy, Pathological complete response, PD-L1 expression, Lymph node status

## Abstract

**Purpose:**

This meta-analysis aims to evaluate the pooled pathological complete response (pCR) rate and the relative benefits across different subgroups in human epidermal growth factor receptor 2 (HER2) -negative breast cancer treated with immunotherapy.

**Methods:**

Clinical trials for neoadjuvant immunotherapy in combination with chemotherapy were identified. The pooled pCR rate of total population and patients across different subgroups was performed.

**Results:**

Totally, the meta-analysis included 15 clinical trials comprising 3,885 patients. The pooled pCR rate was 58% (95% CI, 54–62) for triple-negative breast cancer (TNBC) patients and 25% (95% CI, 22–27) for hormone receptor (HR)-positive/HER2-negative (HR+HER2-) patients, respectively. However, the relative benefit of neoadjuvant chemotherapy combined with immunotherapy compared to chemotherapy was comparable (*p* = 0.70), with odds ratio (OR) of 1.76 (95% CI, 1.43–2.17) in TNBC patients and 1.87 (95% CI, 1.49–2.36) in HR+HER2- patients. Subgroup analysis showed that programmed cell death ligand-1 (PD-L1)-positive patients had higher pCR benefits compared to PD-L1-negative patients, regardless of whether it was immunotherapy combined with neoadjuvant chemotherapy or neoadjuvant chemotherapy alone, with OR of 3.41 (2.61–4.45) and 2.48 (1.80–3.42) respectively. Conversely, among patients receiving chemotherapy alone, the pCR rate in lymph node-positive cases was 42% lower than that in lymph node-negative cases, while the addition of immunotherapy enabled lymph node-positive patients to achieve a comparable pCR rate to lymph node-negative patients (OR, 1.08).

**Conclusion:**

Immunotherapy showed substantial benefits in improving pCR rates in both TNBC and HR+HER2- patients when combined with neoadjuvant chemotherapy, especially in lymph node-positive breast cancer patients.

**Supplementary Information:**

The online version contains supplementary material available at 10.1186/s12885-026-16230-9.

## Introduction

Breast cancer is a main leading cause of cancer-related mortality worldwide [1]. Driven by the desire to make breast-conserving surgery more feasible and to achieve higher cure rates, neoadjuvant chemotherapy (NAC) has been a standard component of the treatment paradigm for early-stage breast cancer. However, it was shown that 24.9% of early-stage breast cancer patients receiving neoadjuvant therapy had a distant recurrence within 5 years [2]. Therefore, there is an urgent need for therapeutic strategies that can further improve patient cure rates. Unlike patients with human epidermal growth factor receptor 2 (HER2) expression who can benefit successful from targeted therapies such as trastuzumab and pertuzumab, HER2-negative breast cancers (hormone receptor-positive and triple-negative breast cancers [TNBC]), which account for a significant proportion of all breast cancer cases, lack a synergistic therapy strategy that can further enhance the efficacy of neoadjuvant chemotherapy. In particular, HR+HER2- breast cancer has limited sensitivity to neoadjuvant chemotherapy, with pCR rate of only 7.5%-16. 2% [2].

Immunotherapy, exemplified by programmed cell death protein-1 (PD-1)/ programmed cell death ligand-1 (PD-L1) inhibitors, has significantly improved the treatment landscape for various malignancies including breast cancer [3]. The long response duration of immunotherapy makes it a significant hope for improving the cure rates of patients. Among all molecular subtypes, TNBC, characterized by its high immunogenicity [4], has demonstrated encouraging responses to immunotherapy, particularly in the neoadjuvant setting [5, 9]. Though once considered as “cold tumor” with poor immunogenicity [10], HR+/HER2- breast cancer has also been proved promising in benefiting from neoadjuvant immunotherapy recently [11, 12], marking an additional watershed moment in the realm of breast cancer immunotherapy.

Despite the advancements in the combination of NAC and immunotherapy for HER2-negative breast cancer, there are still several challenges in its management. For instance, not all patients respond equally to the combination regimes. The heterogeneity of breast cancer subtypes, the complexity of treatment regimens, and the occurrence of treatment resistance pose significant challenges in optimizing patient care. To date, several studies have investigated the efficacy of combining NAC with immunotherapy in HER2-negative breast cancer. These studies have evaluated the pathological complete response (pCR) rate, event-free survival (EFS), and toxicities associated with the use of NAC combined with immunotherapy. However, the results of these studies have been variable, and there is a lack of consensus on the optimal combination of treatments and the patient population that may benefit the most from this novel approach. Accordingly, it is imperative to proceed from evidence-based medical evidence to optimize treatment regimens and identify the patient population that may derive the greatest benefits from this approach.

To provide a comprehensive initial overview of immunotherapy efficacy across the HER2-negative spectrum, this meta-analysis was conducted to assess the pCR rate and immune-related adverse event (irAE) in early-stage triple-negative and HR+/HER2- breast cancers treated with neoadjuvant chemoimmunotherapy. Furthermore, through pre-specified subgroup analyses, we seek to delineate their divergent responses, thereby helping to identify the patient population that may benefit the most from this novel treatment approach to guide future clinical trials and treatment strategies.

## Methods

### Search strategy

The systematic review and meta-analysis were performed following the Preferred Reporting Items for Systematic Reviews and Meta-Analyses (PRISMA) guidelines [13]. A comprehensive search was conducted in the MEDLINE, EMBASE, Cochrane Library databases, as well as in the conference abstracts from the American Society of Clinical Oncology, European Society for Medical Oncology, and San Antonio Breast Cancer Symposium within the past two years, for all English-language records published from January 2011 to March 2024. The search terms were: “pembrolizumab” or “atezolizumab” or “nivolumab” or “durvalumab” or “avelumab” or “cemiplimab” or " camrelizumab” or " sintilimab” or " toripalimab” or " tislelizumab” or “programmed cell death 1” or “programmed cell death ligand 1” or “PD-1” or “PD-L1” or “immune checkpoint” or “immunotherapy” and “breast cancer” or “breast carcinoma” or " breast neoplasms” or “HER2 negative” or “TNBC” or “ER+HER2-” and “early” or “neoadjuvant” or “preoperative”. The inclusion criteria for the study were as follows: (1) prospective studies, (2) patients diagnosed with early-stage HER2 negative breast cancers (TNBC or HR+HER2-) confirmed by histology and cytology, (3) experimental intervention group receiving PD-1/PD-L1 inhibitors in combination with chemotherapy, (4) clinical trials that reported pCR data.

### Data extraction

Two independent researchers (WYH and QYL) selected relevant articles and extracted data for quality assessment. Discrepancies were adjusted and consensus was reached through the discussion with a third expert. For each included trial, we extracted basic information about the study (trial name, first author, publication year, number of patients enrolled), baseline clinical and pathological characteristics of patients, as well as types and combinations of chemotherapy and checkpoint inhibitors, etc. The primary endpoint of our study was pCR, defined as the absence of invasive tumors in the breast and local lymph nodes at the time of surgery. Therefore, we separately extracted pCR data for both the intention-to-treat population and predefined subgroups including PD-L1 expression, T stage, N stage, stage, age, Eastern Cooperative Oncology Group (ECOG) score, etc. Additionally, we extracted the occurrences of any grade and grade 3 or above irAE in patients from each clinical trial, as well as the occurrences of specific adverse events of interest, as our secondary study endpoint.

### Statistical analysis

Firstly, we conducted an overall meta-analysis for all included clinical trials, comprehensively reporting the rates of pCR and irAE, defined respectively as the number of patients experiencing pCR and irAE divided by the total number of patients. For the pCR data concerning various baseline characteristics of patients within each clinical trial, pooled analyses were performed separately, and interaction tests were conducted to assess the differences in efficacy among these subgroups. The weight of each study was calculated using the inverse variance method; the pooled rate was presented with 95% confidence interval (CI). Meta-regression was performed for different combinations in different clinical trials to study the optimal impact of each element.

Building upon this foundation, the data from randomized controlled trials (RCTs) was further analyzed. We separately conducted pooled analyses of pCR data for different subgroups of patients in both the experimental and control groups, and the results were presented with odds ratios (OR) with 95% CI. Interaction tests were performed to evaluate the differences in efficacy between the two treatment groups.

The *I*^2^ test was used to evaluate inter-study heterogeneity: *p* < 0.1 or *I*^2^ > 50% indicating significant heterogeneity, thus random-effects model being used. Conversely, *p* > 0.1 or *I*^2^ < 50% indicated no significant heterogeneity, and a fixed-effects model was used. All tests were two-sided, and *p* < 0.05 was considered statistically significant. All statistical analyses and forest plots were performed using Stata SE 16 software (StataCorp, College Station, TX, USA).

## Results

**Fig. 1:**
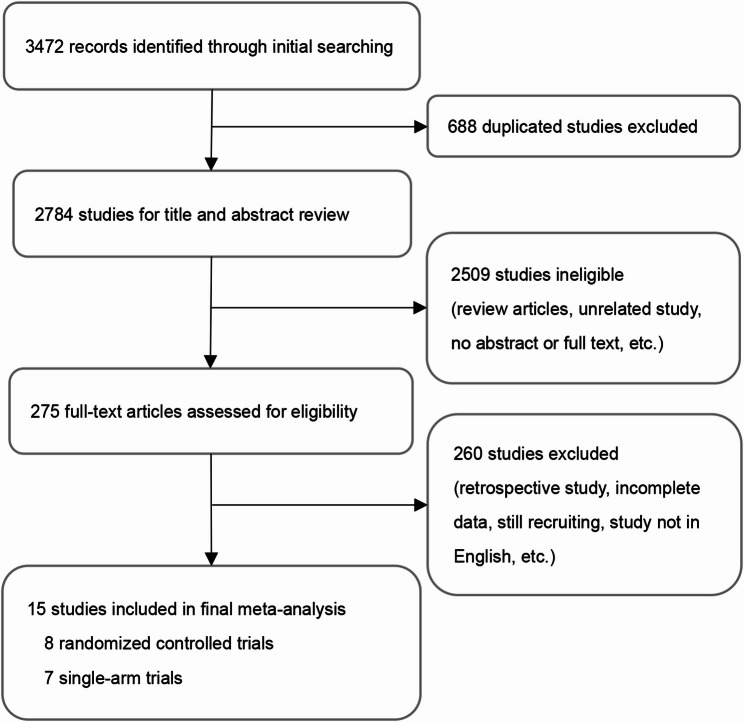
Flow diagram of study selection

The initial literature search yielded a total of 3,472 records. After screening titles, abstracts, and full-texts, we ultimately identified 15 clinical trials, consisting 8 randomized controlled trials [5, 9, 11, 12, 14, 17] and 7 single-arm clinical trials [18, 23]. The results of the review process are summarized in Fig. 1. All studies were prospective and were published between 2020 and 2023. Among these, 12 clinical trials focused on TNBC research [5, 7, 9, 14, 23] and were reported by full-article, while 2 were related to HR+HER2- patients and were presented by conference abstract [11, 12]. The remaining I-SPY2 trial included HER2-negative patients, therefore divided into two separate records (TNBC and HR+HER2-) for statistical analysis [8]. Consequently, a total of 16 records of clinical trials were included in the study. PD-1 inhibitors were evaluated in 11 records [8, 9, 11, 12, 14, 18, 21, 23], and PD-L1 inhibitors were examined in 5 records [5, 7, 15, 17, 22]. The meta-analysis encompassed a total of 3,885 patients, of whom 2195 received immunotherapy combination and 1690 underwent neoadjuvant chemotherapy alone. The main characteristics of the included trials are summarized in Table 1.

**Table 1:** Summary of the 16 records of studies evaluated in the meta-analysis

Trial								Baseline (%)			
	Phase	Trial type	Tumor subtype	Stage	Treatment regime	Sample	pCR (%)	*N*+	T3-T4	stage III	PD-L1+
KEYNOTE-522	III	RCT	TNBC	II-III	Pembrolizumab+(TCb*4-AC*4)	401	64.8	51.7	26	24.7	83.7
IMpassion031	III	RCT	TNBC	II-III	Atezolizumab+(T*4-AC*4)	165	58	34	30	22	47
NeoTRIP	III	RCT	TNBC	II-III	Atezolizumab + TCb*8	138	48.6	87	46	50	57
I-SPY2 (TNBC)	II	RCT	TNBC	II-III	Pembrolizumab+(T*4-AC*4)	29	60	-	-	-	-
GeparNuevo	II	RCT	TNBC	I-III	Durvalumab+(T*4-EC*4)	88	53.4	31.4	7.9	-	88.5
NCI 10,013	II	RCT	TNBC	II-III	Atezolizumab + TCb*4	45	55.6	46.7	-	31.1	35.6
KEYNOTE-756	III	RCT	HR+HER2-	II-III	Pembrolizumab+(T*4-AC*4)	635	24.3	89.8	36.7	-	75.9
CheckMate-7FL	III	RCT	HR+HER2-	II-III	Nivolumab+(T*4-AC*4)	257	24.5	80	-	46	34
I-SPY2 (HR+HER2-)	II	RCT	HR+HER2-	II-III	Pembrolizumab+(T*4-AC*4)	40	30	-	-	-	-
NeoPACT	II	single-arm	TNBC	I-III	Pembrolizumab + TCb*6	111	58	39	18	13	46
KEYNOTE-173	Ib	single-arm	TNBC	II-III	Pembrolizumab+(T*4-AC*4)	60	60	67	26	25	78
J. Foldi, et al., 2021	Ib/II	single-arm	TNBC	I-III	Durvalumab+(T*4-AC*4)	55	44	48	14	-	56
NeoImmunoboost	II	single-arm	TNBC	I-III	Pembrolizumab+(T*4-AC*4)	50	66	30	6	-	84.8
CTRIO	II	single-arm	TNBC	II-III	Tislelizumab + TCb*6	62	56.5	74.2	24.2	32.3	61
C. Zheng, et al., 2024	II	single-arm	TNBC	II-III	Camrelizumab + AT*6	39	64.1	56.4	2.6	-	30.8
C. Wang, et al., 2023	II	single-arm	TNBC	II-III	Camrelizumab+(T*4-AC*4)	20	65	69.6	4.3	-	-

*Abbreviations*: *TNBC* Triple-negative breast cancer, *HR* Hormone receptor, *HER2* Human epidermal growth factor receptor 2, *RCT* Randomized controlled trial, *A* doxorubicin/epirubicin, *C* cyclophosphamide, *T* taxane, *Cb* carboplatin, *pCR* pathological complete response, *PD-L1* programmed cell death ligand-1

"*" stands for "×" (cycles)

**Fig. 2:**
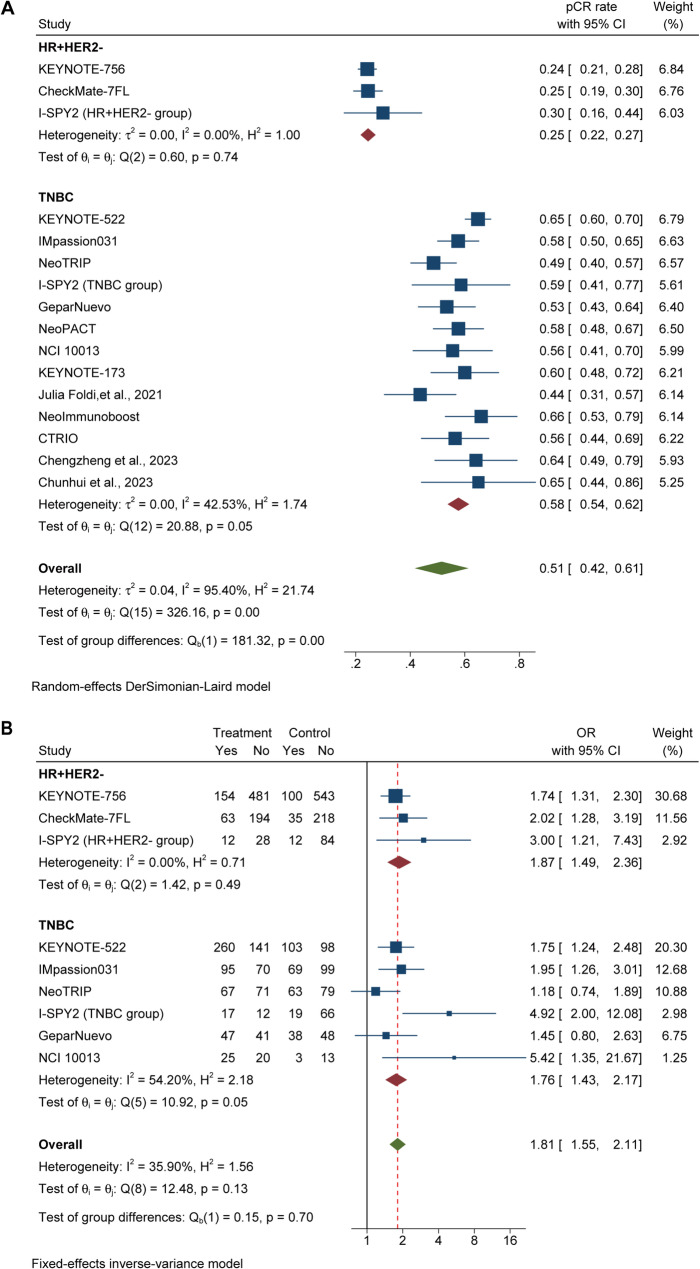
Forest plots of (**A**) pooled pCR rates, (**B**) pooled odds ratios for pCR between immunotherapy combination and chemotherapy group, in patients with HR+HER2- and TNBC subtypes

In total, the pooled pCR rate was 51% (95% CI, 42–61), with half of the patients achieving pCR within the study’s follow-up period (Fig. 2A). Significant differences were observed in pCR rates among patients with different molecular subtypes (*p* < 0.01), with TNBC patients exhibiting a pooled pCR rate of 58% (95% CI, 54–62), whereas HR+HER2- patients had a considerably lower rate of 25% (95% CI, 22–27). Intriguingly, comparative analysis showed that the relative benefits from the combination group versus chemotherapy were comparable across both subtypes (subgroup interaction, *p* = 0.70). Specifically, the combination of immunotherapy with neoadjuvant chemotherapy significantly increased the pCR rate by 76% (OR, 1.76; 95% CI, 1.43–2.17) for TNBC patients and by 87% (OR, 1.87; 95% CI, 1.49–2.36) for HR+HER2- patients (Fig. 2B).

**Fig. 3:**
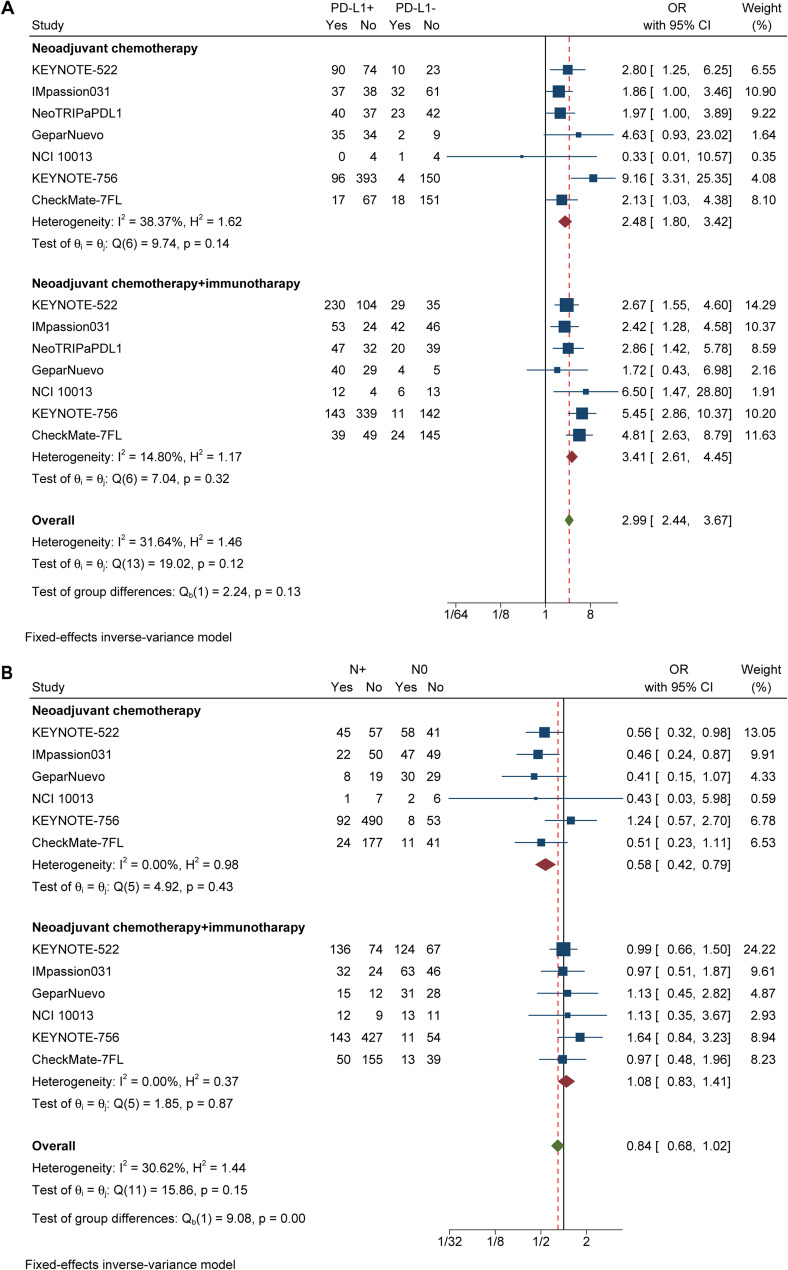
Forest plots of odds ratios for pCR between (**A**) PD-L1 expression positive and negative, (**B**) Node involvement positive and negative, in patients treated with immunotherapy combination and chemotherapy, respectively

The subgroup analysis of single-arm trials on neoadjuvant immunotherapy revealed that PD-L1-positive patients achieved higher rates of pCR. For TNBC, the pCR rates were 68% (95% CI, 63–73) for PD-L1-positive patients and 41% (95% CI, 0.36–0.46) for PD-L1-negative patients. For HR+/HER2- breast cancer patients, the pCR rates were 36% (95% CI, 22–50) and 10% (95% CI, 4–17) respectively. These differences were both statistically significant (*p* < 0.01) (Table 2; Figure S1-S2). However, subgroup analyses of experimental and control groups in RCTs revealed that regardless of whether it was immunotherapy combined with neoadjuvant chemotherapy or neoadjuvant chemotherapy alone, PD-L1-positive patients had higher pCR benefits compared to PD-L1-negative patients, with OR of 3.41 (95% CI, 2.61–4.45) and 2.48 (95% CI, 1.80–3.42) respectively, and the difference in efficacy between the two treatment strategies was not statistically significant (interaction, *p* = 0.13; Fig. 3A).

**Table 2:** Pooled pathological complete response among different subgroups

Subgroup	TNBC			HR+HER2-		
	No. of trials	pCR (95%CI)	*P* (interaction)	No. of trials	pCR (95%CI)	*P* (interaction)
PD-L1	10		< 0.01	2		< 0.01
PD-L1+		0.68 (0.63–0.73)			0.36 (0.22–0.50)	
PD-L1-		0.41 (0.36–0.46)			0.10 (0.04–0.17)	
N stage	8		0.7	2		0.22
N+		0.59 (0.55–0.64)			0.25 (0.22–0.28)	
N0		0.60 (0.56–0.65)			0.20 (0.13–0.27)	
T stage	4		0.37	1		< 0.01
T1-T2		0.57 (0.45–0.69)			0.28 (0.23–0.32)	
T3-T4		0.51 (0.42–0.59)			0.19 (0.14–0.23)	
Stage	4		0.01	1		0.54
II		0.65 (0.61–0.70)			0.23 (0.16–0.30)	
III		0.53 (0.46–0.61)			0.26 (0.18–0.34)	
Age	4		0.35	-		-
<40		0.60 (0.51–0.69)			-	
≥40		0.55 (0.49–0.61)			-	
ECOG	2		0.81	1		0.21
0		0.63 (0.59–0.67)			0.25 (0.21–0.29)	
1		0.62 (0.51–0.72)			0.19 (0.09–0.28)	

*Abbreviations*: *TNBC* Triple-negative breast cancer, *HR* Hormone receptor, *HER2* Human epidermal growth factor receptor 2, *pCR* Pathological complete response, *PD-L1* Programmed cell death ligand-1

Subgroup analysis based on lymph node status revealed that among patients receiving chemotherapy alone, the pCR rate in lymph node-positive cases was 42% lower than that in lymph node-negative cases (OR, 0.58; 95% CI, 0.42–0.79). However, the addition of immunotherapy enabled lymph node-positive patients to achieve a comparable pCR rate to lymph node-negative patients (OR, 1.08; 95% CI, 0.83–1.41). The difference was significant (*p* < 0.01), as depicted in Fig. 3B. Pooled analysis of all clinical trials of neoadjuvant immunotherapy also showed consistent results. The pCR rates for lymph node-positive and negative TNBC patients were 0.59 (95% CI, 0.55–0.64) and 0.60 (95% CI, 0.56–0.65) respectively, while for HR+/HER2- patients, they were 0.25 (95% CI, 0.22–0.28) and 0.20 (95% CI, 0.13–0.27) respectively. The difference in pCR rates between patients with and without lymph node metastasis in both subtypes was not statistically significant (*p* = 0.7; *p* = 0.22; Table 2, Figure S3-S4).

Conversely, analysis based on T stage revealed that regardless of whether it was immunotherapy combined with neoadjuvant chemotherapy or neoadjuvant chemotherapy alone, patients with T3-T4 stage had poorer pCR compared to patients with T1-T2 stage, with odds ratios of 0.51 (95% CI, 0.38–0.68) and 0.58 (95% CI, 0.40–0.84) respectively. The difference in efficacy between the two treatment strategies was not statistically significant (interaction, *p* = 0.58; Figure S5-S6; Table 2). Further exploratory analyses for stage, age, and ECOG score are presented in the appendix (Table 2, Figure S7-S9).

In addition, for single-arm trials and RCTs, we also conducted meta-regression analyses on different combination strategies in various clinical trials to study the optimal impact of each element. The multivariate analysis of single-arm trials found that PD-1 inhibitors seemed to show a higher pCR rate compared to PD-L1 inhibitors, which were 63% (95% CI, 59–66) and 53% (95% CI, 48–57), respectively (Table S1; Figure S10). However, no positive results were found in the analysis of the relative benefit rate in RCTs (Table S2). Other factors such as the type of paclitaxel, whether carboplatin was used, and whether anthracyclines were involved did not show statistical significance (Table S1-S2).

**Fig. 4:**
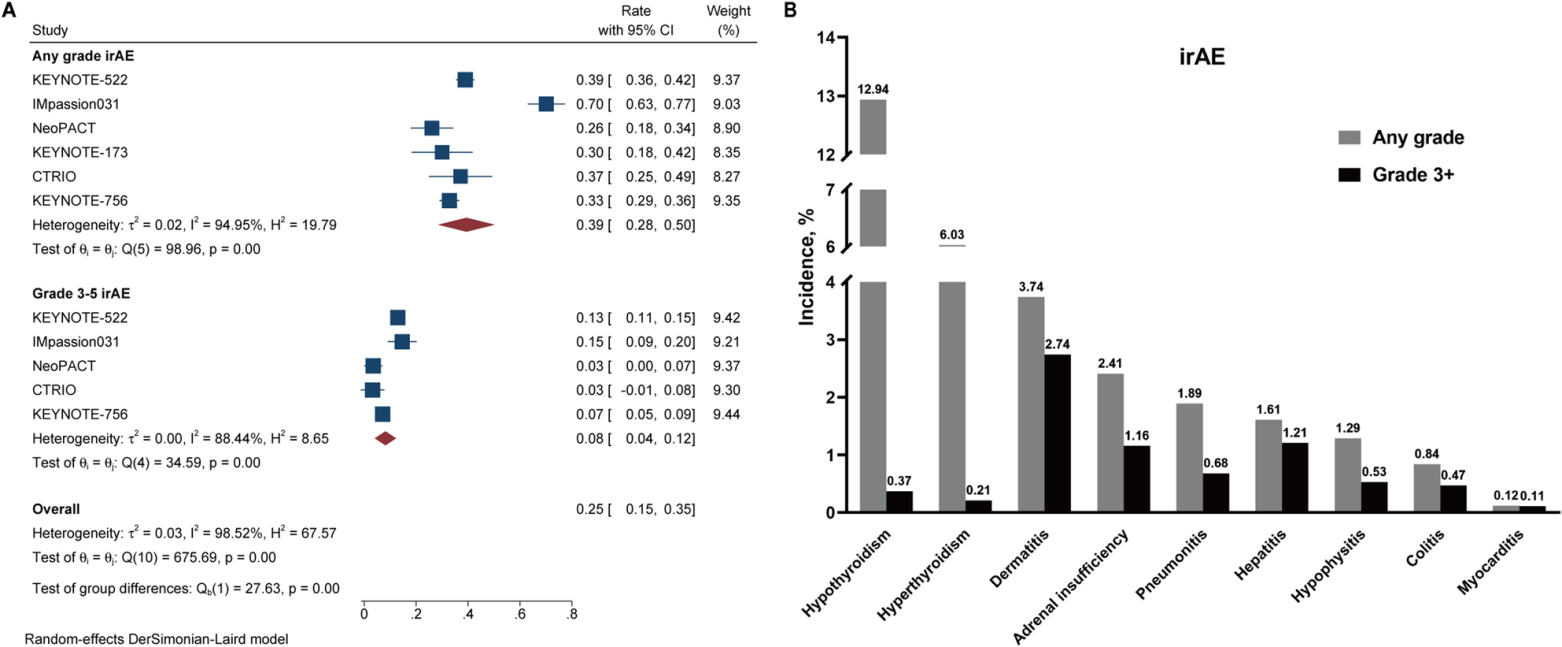
**A** Forest plots of pooled rates of all immune-related adverse effects with any grade and grade 3 or above, and (**B**) bar graph of the incidence rates of specific immune-related adverse effects with any grade and grade 3 or above

Finally, we also statistically analyzed the incidence of irAE in patients receiving neoadjuvant immunotherapy. The incidence rates of any grade irAE and grade 3 or above irAE were 39% (95% CI, 28–50) and 8% (95% CI, 4–12), respectively (Fig. 4A). Specific common grade any and grade 3 or above immune-related adverse events were also tabulated and displayed (Fig. 4B).

## Discussion

This meta-analysis confirmed that the combination of neoadjuvant immunotherapy and chemotherapy significantly improved pCR rate in patients with HER2-negative breast cancer. Although a significant difference exists in pCR rates between patients with TNBC patients and HR+HER2- subtypes, the relative benefits of combining immunotherapy with neoadjuvant chemotherapy is comparable, resulting in a significant improvement in pCR rate by 76% and 87%, respectively. The results of our study bolster the existing evidence of the use of immunotherapy in HER2-negative patients, encompassing both TNBC and HR+HER2- patients. One of the main highlights of our study, compared to previous reports, is the incorporation of the most recently reported data for HER2- patients, revealing that these two subtypes of breast cancers can achieve a comparable benefit rate from neoadjuvant immunotherapy. Subsequent subgroup analysis also indicated a consistent trend across the two molecular subtypes. Indeed, the superiority of adding immunotherapy in early-stage TNBC has been confirmed in terms of both mechanisms and clinical data. However, it was previously believed that hormone receptor-positive breast cancers, characterized by low immunogenicity, may not derive benefit from immunotherapy [10]. This analysis to some extent provides evidence for the use of immunotherapy in patients of this subtype. However, it is noteworthy that the evidence for immunotherapy in HR+/HER2- disease remains limited and preliminary, being based on only a small number of studies (*n* = 3), and longer-term EFS data are still required to further substantiate the foundation. In fact, identifying populations that would benefit most from immunotherapy combination will be a focal point of further investigation, and we have also made a series of attempts based on existing evidence.

Initially, we investigated the impact of PD-L1 expression on neoadjuvant immunotherapy for HER2-negative breast cancer. Preliminary analysis indicated that, consistent with prior knowledge, PD-L1 was indeed associated with a higher pCR rate. To further elucidate its role, we performed comparative analyses of its effects across various treatments and ascertained that patients with positive PD-L1 expression exhibited higher pCR rates irrespective whether they received immunotherapeutic combination or monochemotherapy. In other words, PD-L1 expression seems to function more as a prognostic indicator than a predictive factor for neoadjuvant immunotherapy in HER2-negative breast cancer. There are numerous hypotheses to explain the results. It is will-known that besides tumor cells, PD-L1 is expressed by various cell types, including macrophages, dendritic cells, T cells, B cells, and endothelial cells [24]. One study have demonstrated that 89% of breast cancers with positive PD-L1 expression exhibited significant tumor-infiltrating lymphocytes (TILs) infiltration, whereas it was only 24% for PD-L1 negative cancers [25]. Multiple studies have confirmed that TILs serve as a robust prognostic biomarker in some early breast cancer subtypes [26]. Therefore, PD-L1 positivity often indicates higher immune infiltration, thus predicting patient prognosis and response to immunotherapy. Nevertheless, at the very least, it is understood that for patients seeking downstaging through neoadjuvant treatment, PD-L1 expression can serve as a crucial guiding indicator, particularly for those with HR+HER2-negative subtypes who may not easily benefit from neoadjuvant treatment.

Furthermore, our research indicates that lymph node metastasis can serve as a crucial consideration in determining the addition of immunotherapy. It was observed that for neoadjuvant chemotherapy alone, pCR rates in node-positive patients were only 58% of those in node-negative patients, while immune combination therapy enabled lymph node-positive patients to achieve similar pCR rates to lymph node-negative patients, regardless of breast cancer molecular subtypes. Conversely, analysis of T stage revealed that both immunotherapy combination and neoadjuvant chemotherapy alone resulted in poorer pCR rates for patients with T3-T4 stage compared to those with T1-T2 stage. These results suggest that immunotherapy appears to excel in eliminating more lymph node metastatic lesions compared to controlling the primary tumor. Lymph node metastasis is recognized as a significant adverse prognostic factor in early breast cancer, positioning immunotherapy as a beacon of hope for these patients. The role of lymph nodes in immunotherapy is gaining increasing attention, with multiple studies revealing its indispensable role in normal anti-tumor immunity and immune therapeutic efficacy [27, 29]. Tumor-draining lymph nodes are a potent source of anti-tumor immunity, making them a potential target for successful immunotherapy [30, 32]. Several studies have revealed the unique immune suppressive microenvironment within metastatic lymph nodes, characterized by increased exhaustion spectrum of CD8 + T cells, indicating reduced effector potential and elevated expression of checkpoint molecules such as CD39, T cell immunoglobulin domain and mucin domain-3, PD-L1, etc [33, 34]. Consequently, for patients with metastatic lymph nodes, chemotherapy exhibits significantly poorer efficacy, necessitating immunotherapy to reverse this immune-suppressed state. However, it is noteworthy that while there is ample evidence supporting the use of cyclin-dependent kinase 4/6 (CDK4/6) inhibitors in adjuvant therapy for high-risk HR+/HER2-negative early breast cancer with lymph node positivity, there is a notable level of toxicity overlap between CDK4/6 inhibitors and immunotherapy [35, 36]. Therefore, managing adverse effects and selecting optimal candidates for neoadjuvant immunotherapy are questions worthy of further exploration and consideration.

Undoubtedly, our meta-analysis possesses certain limitations. The primary limitation is the lack of individual patient-level data. Despite our comprehensive efforts to source data from various stages of publication including journals, appendices, and conference abstracts for each clinical trial, our access was limited to published data only. Consequently, our analysis relied solely on the accessible published data, and certain subgroup data may exhibit some bias due to limited sample sizes, thus requiring cautious interpretation of some points. Furthermore, although pCR has been established to hold significant statistical significance with survival, recent studies have raised questions about its predictive efficacy [37]. However, due to the scarcity of clinical trials with survival data in this domain, the limited number of trials may lead to substantial biases. Consequently, in this paper, we focused our detailed analysis solely on pCR. Even so, the recent results from KEYNOTE-522 demonstrate that the pCR benefit from neoadjuvant pembrolizumab plus chemotherapy can translate into a significant survival improvement [38]. We expect that upcoming clinical trials with relevant data will provide more extensive EFS data, thus offering further insights into long-term prognosis.

In conclusion, our data demonstrated that the combination of neoadjuvant chemotherapy and immunotherapy significantly improved pCR rate in HER2-negative patients, with TNBC and HR+/HER2- patients showing comparable levels of improvements. Compared to PD-L1 expression, lymph node status may be a crucial consideration in guiding treatment selection.

## Supplementary Information

Supplementary Material 1.

## Data Availability

The datasets used and/or analyzed during the current study are available from the corresponding author on reasonable request.

## Abbreviations

CDK4/6: Cyclin-dependent kinase 4/6
CI: Confidence interval
ECOG: Eastern Cooperative Oncology Group
EFS: Event-free survival
HER2: Human epidermal growth factor receptor 2
HR: Hormone receptor
irAE: Immune-related adverse event
NAC: Neoadjuvant chemotherapy
OR: Odds ratio
pCR: Pathological complete response
PD-1: Programmed cell death protein-1
PD-L1: Programmed cell death ligand-1
PRISMA: Preferred Reporting Items for Systematic Reviews and Meta-Analyses
RCT: Randomized controlled trial
TILs: Tumor-infiltrating lymphocytes
TNBC: Triple-negative breast cancer
