# Supplementary material for: Meta-analysis reveals pathological complete response benefits from neoadjuvant immuno-chemotherapy combination in patients with HER2-negative breast cancer
Source: BMC Cancer. 2026 Jun 8;26:938. doi: 10.1186/s12885-026-16230-9 (PMC13445683; doi:10.1186/s12885-026-16230-9)

Supplementary Materials

Table S1 Results of meta-regressions for pooled pCR by the treatment element of each trial

Covariate for pooled pCR	Estimate (95% CI)	p
platinum (with platinum vs without platinum)	-0.01 (-0.12, 0.11)	0.92
taxane (nab-paclitaxel vs paclitaxel)	-0.02 (-0.10, 0.06)	0.57
anthracycline (with anthracycline vs without anthracycline)	0.04 (-0.91, 0.17)	0.53
regime (anti-PD-L1 vs anti-PD-1)	-0.09 (-0.17, -0.01)	0.03
subtype (HR+HER2- vs TNBC)	-0.41 (-0.52, -0.29)	0.00

Table S2 Results of meta-regressions for pooled odds ratio of pCR by the treatment element of each trial

Covariate for odds ratio of pCR	Estimate (95% CI)	p
platinum (with platinum vs without platinum)	-0.13 (-2.59, 0.53)	0.13
taxane (nab-paclitaxel vs paclitaxel)	-1.52 (-3.90, 0.86)	0.13
anthracycline (with anthracycline vs without anthracycline)	-0.64 (-2.47, 1.19)	0.35
regime (anti-PD-L1 vs anti-PD-1)	0.49 (-2.36, 3.34)	0.62
subtype (HR+HER2- vs TNBC)	-0.97 (-2.47, 0.54)	0.13

Fig S1 Pooled pCR of different PD-L1 expression in TNBC patients.

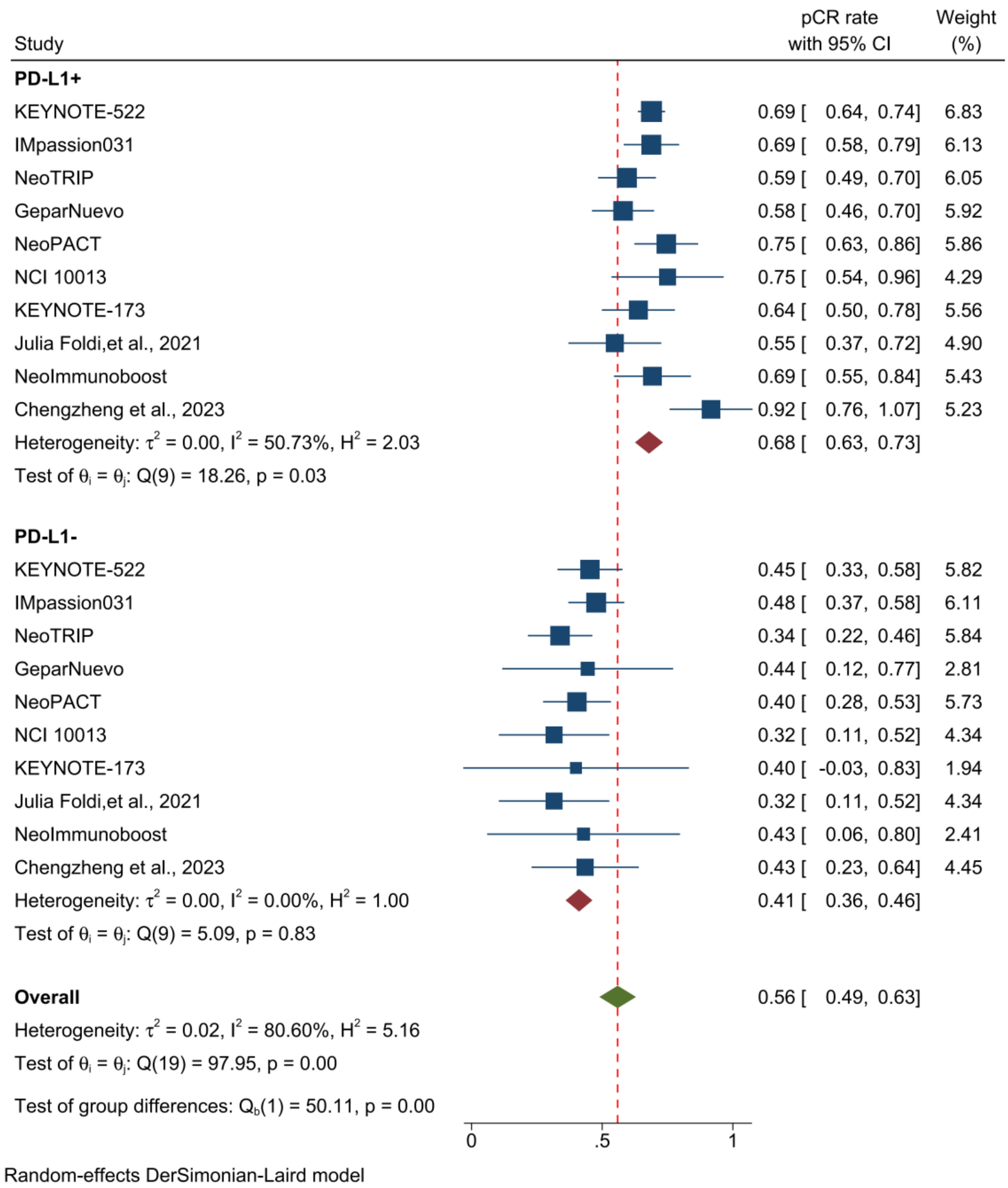

Fig S2 Pooled pCR of different PD-L1 expression in HR-positive HER2-negative breast cancer.

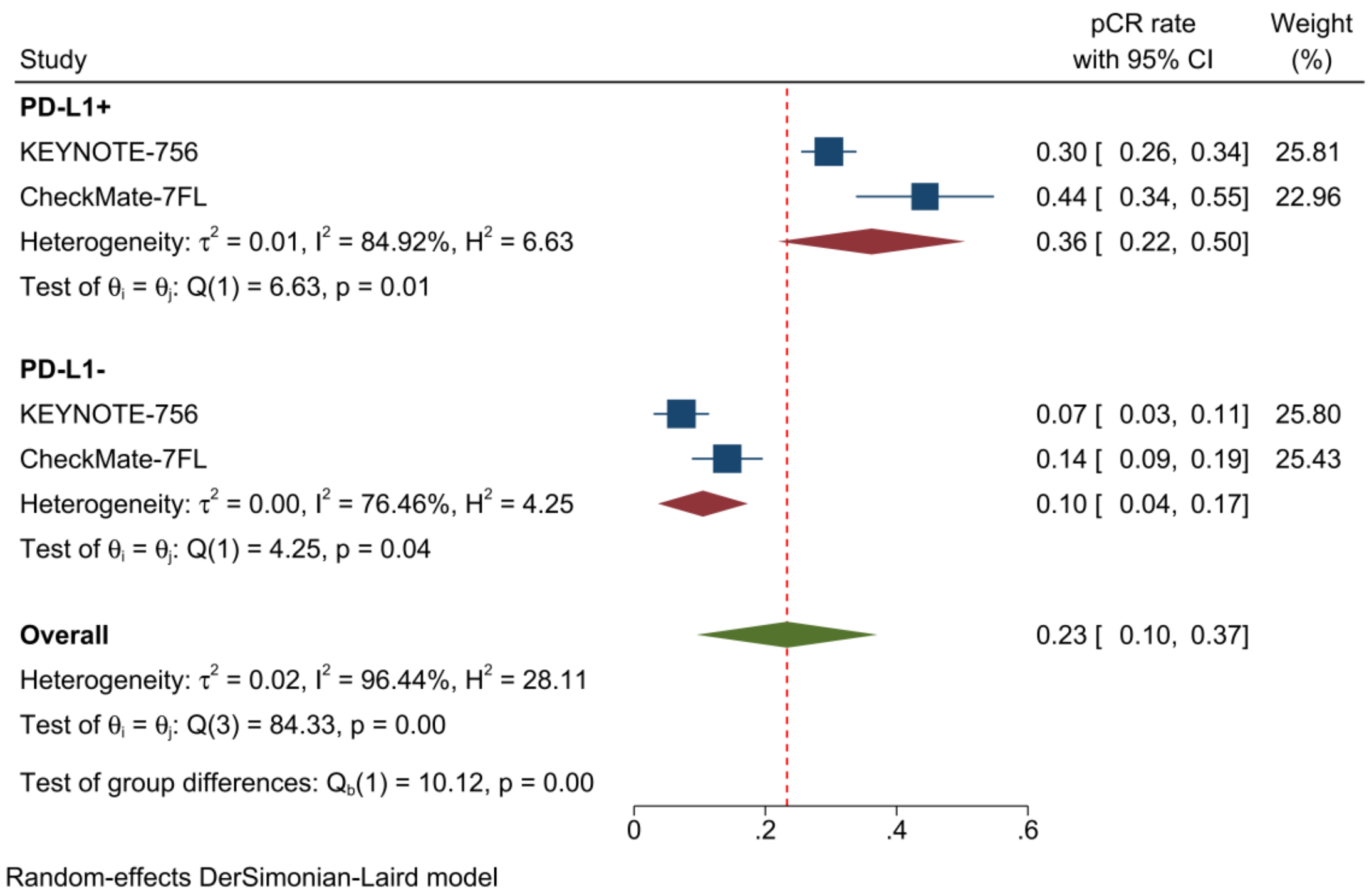

Fig S3 Pooled pCR of different node state in TNBC patients.

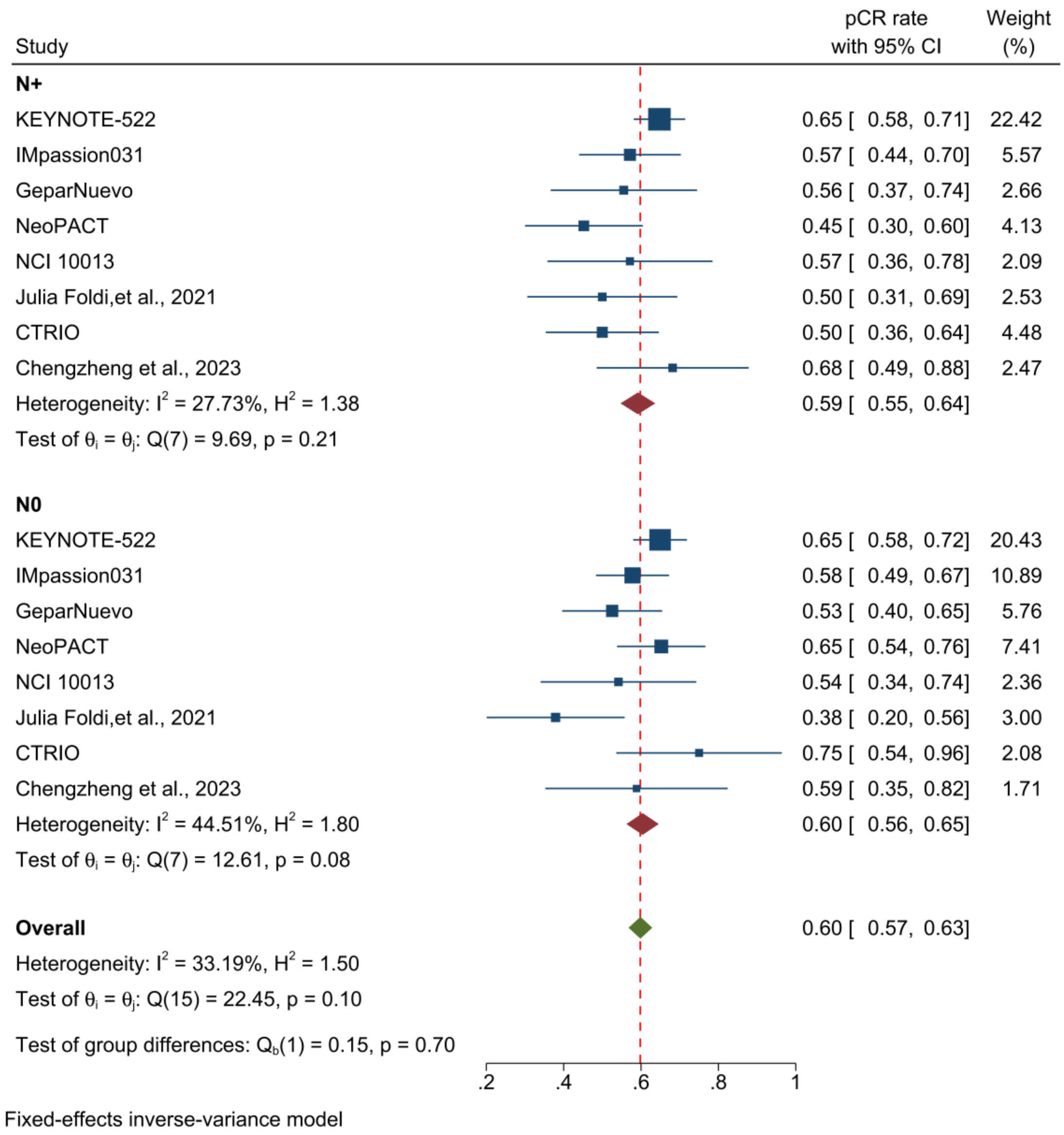

Fig S4 Pooled pCR of different node state in HR-positive HER2-negative breast cancer.

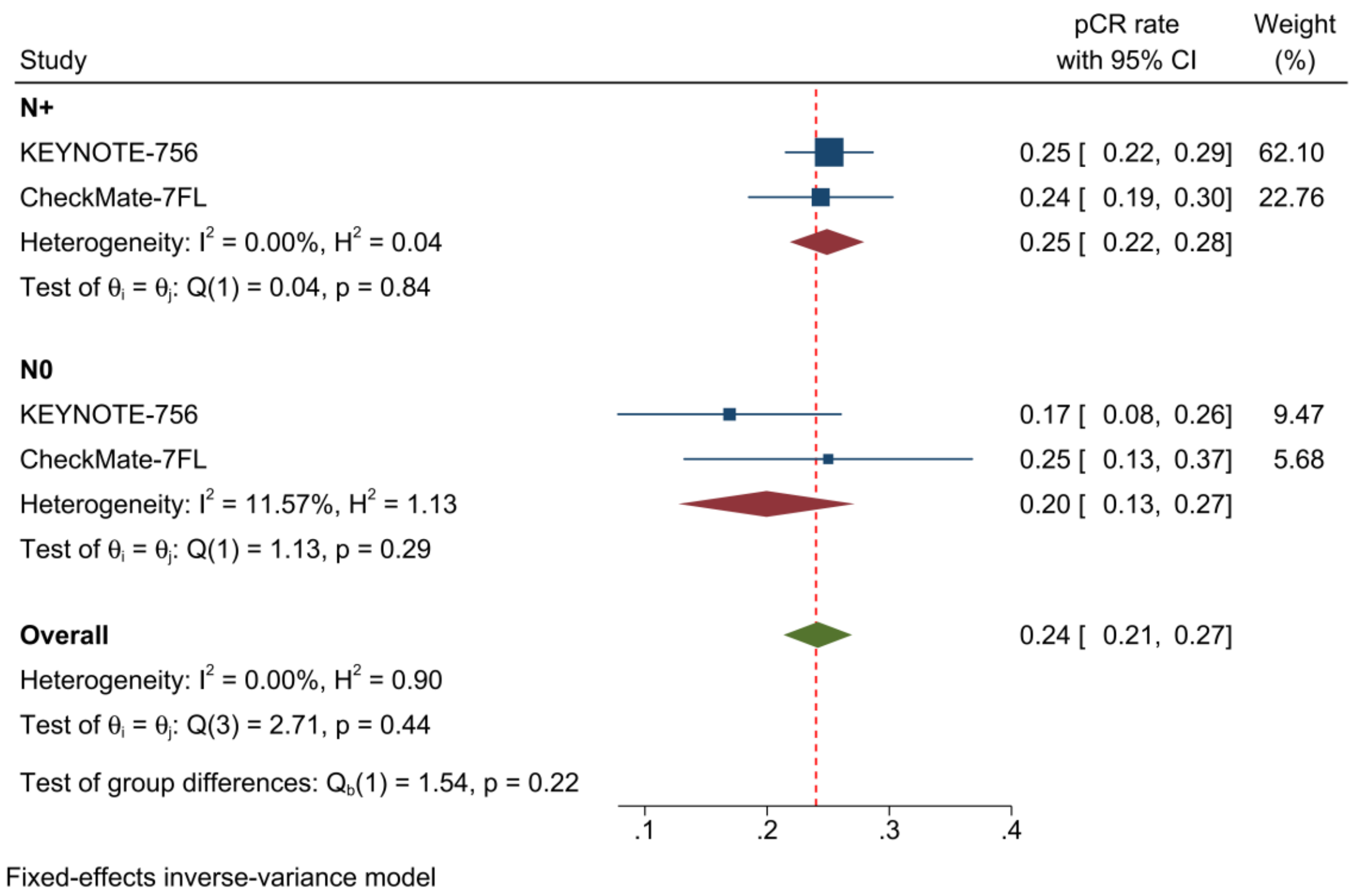

Fig S5 Pooled odds ratio for pCR of different tumor stage in HER2 negative patients.

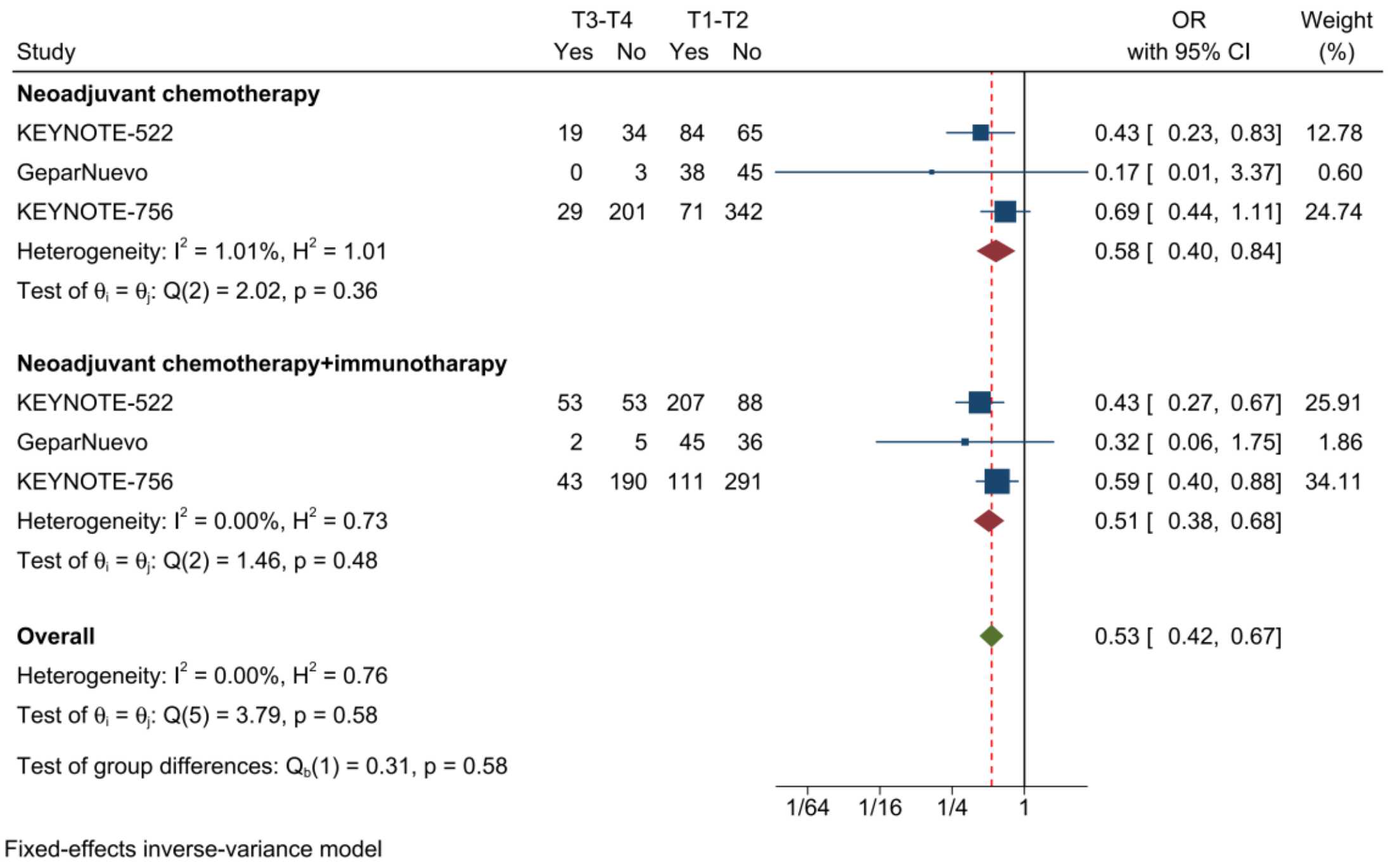

Fig S6 Pooled pCR of different tumor stage in TNBC patients.

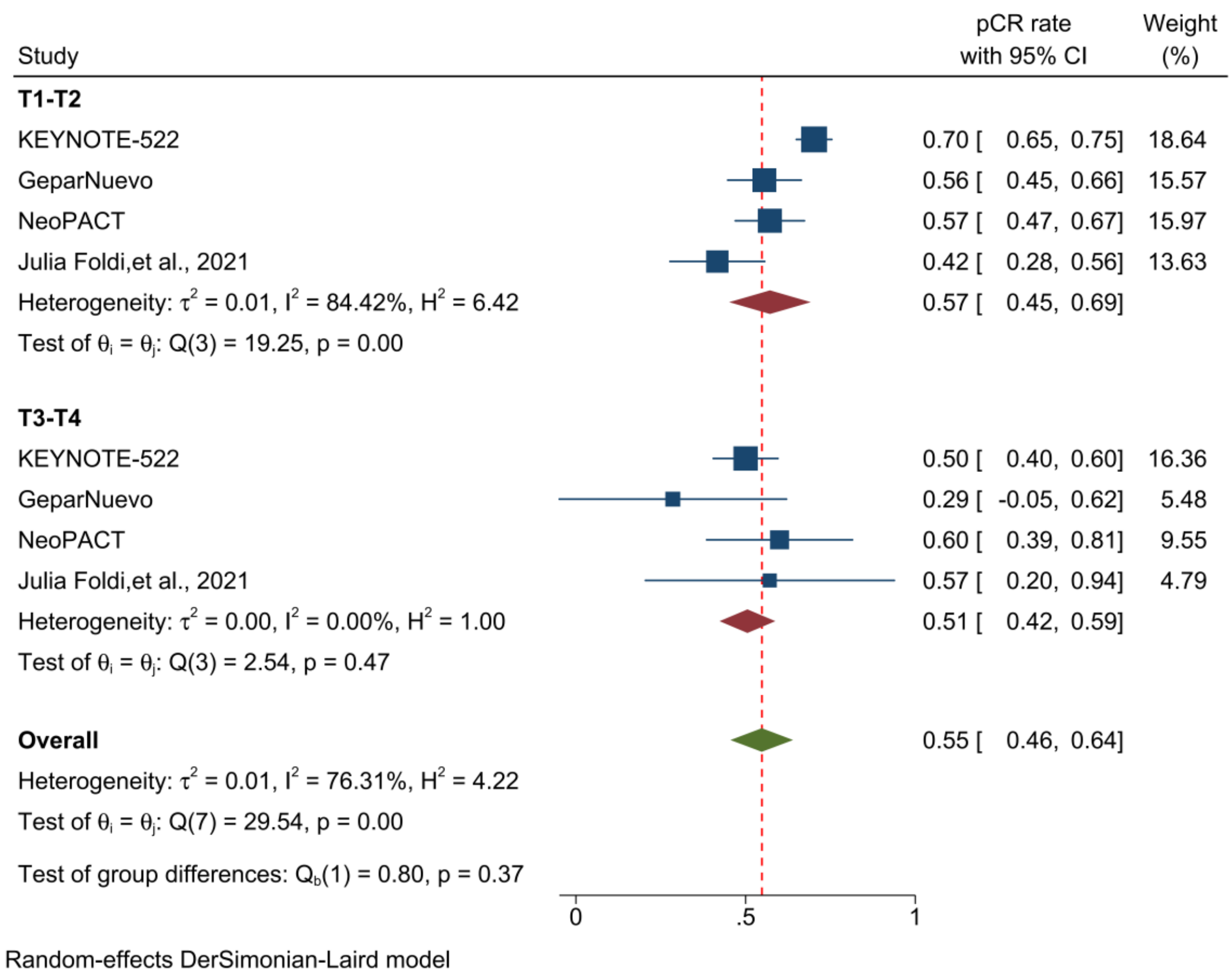

Fig S7 Pooled pCR of different stage in TNBC patients.

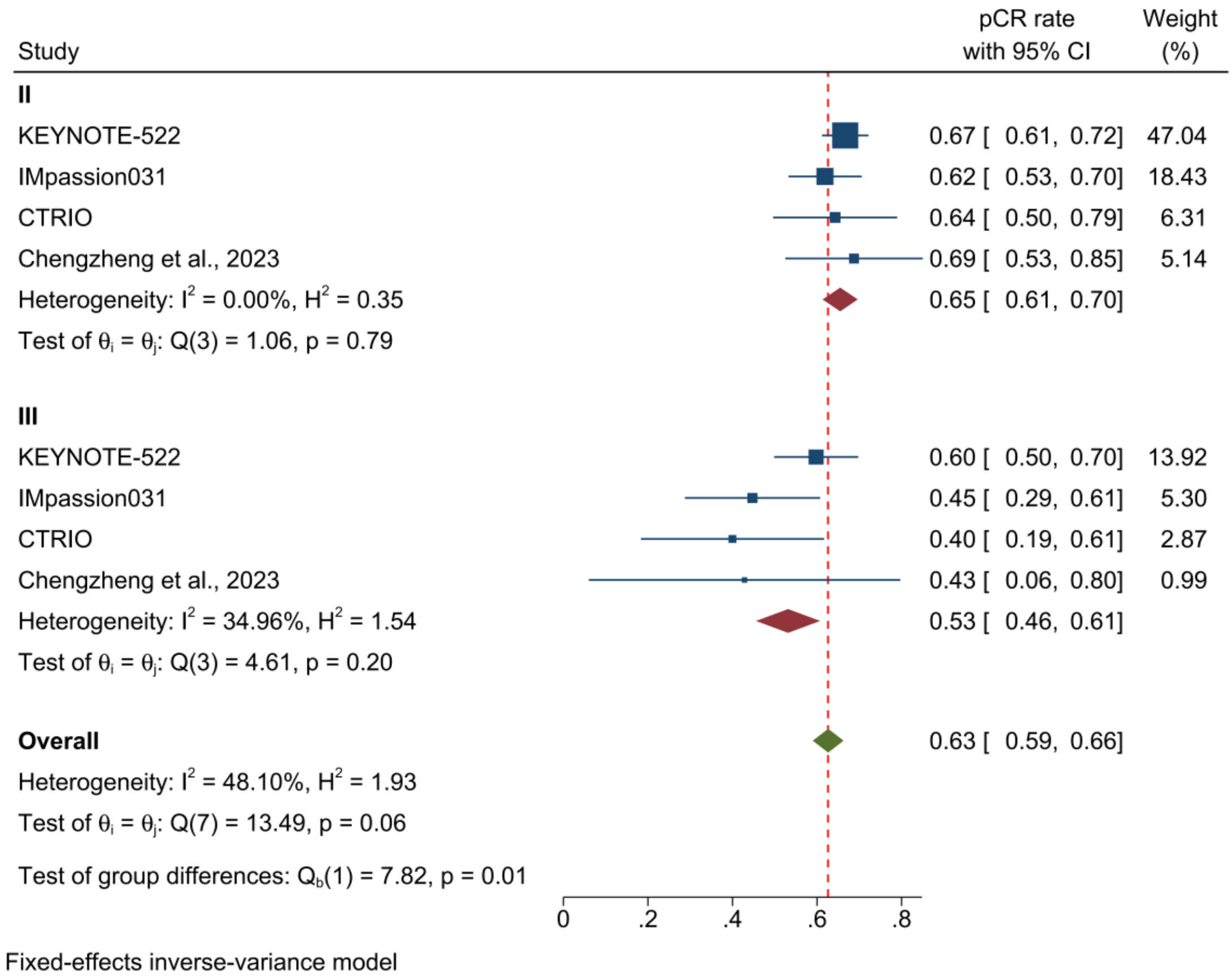

Fig S8 Pooled pCR of different age group in TNBC patients.

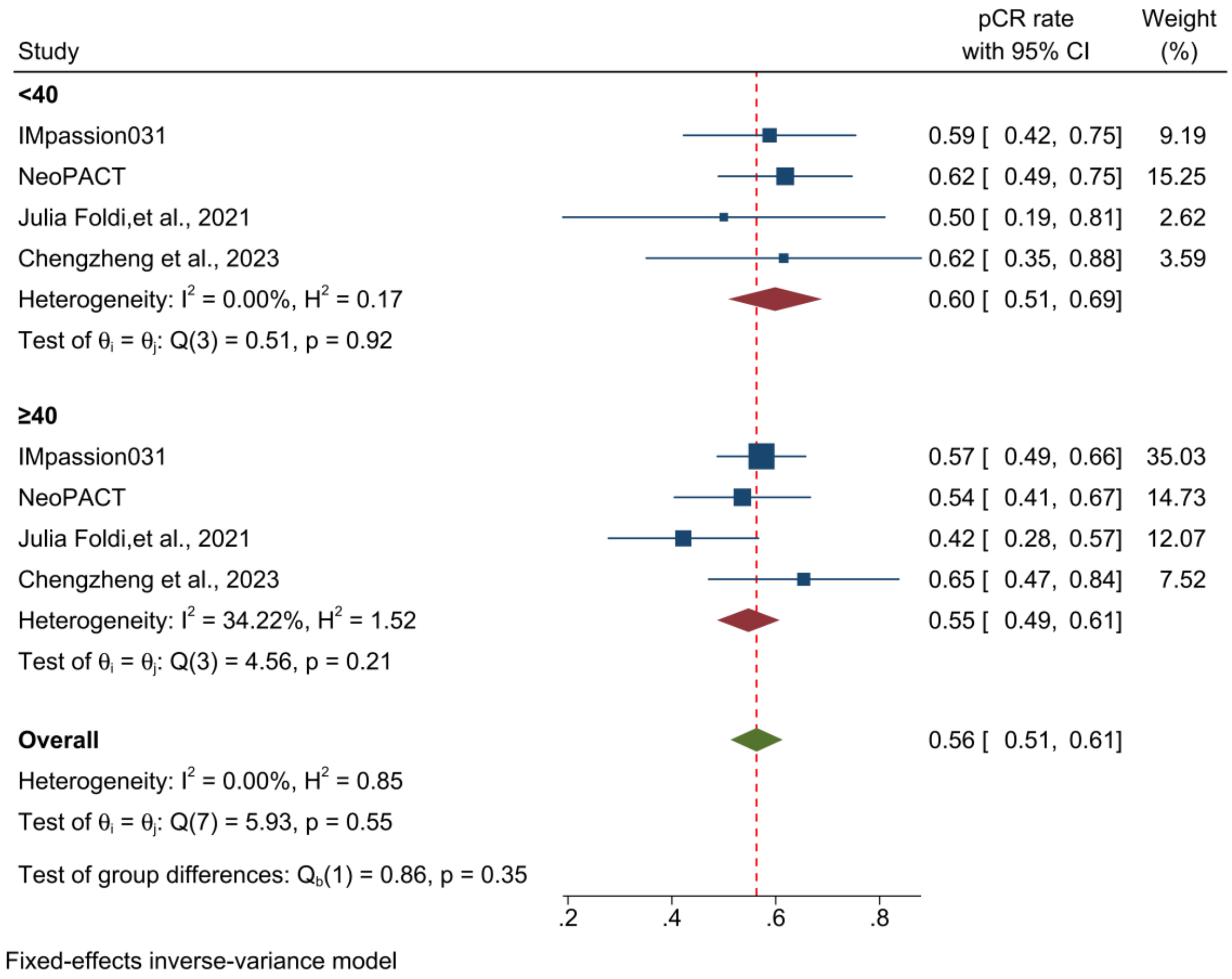

Fig S9 Pooled pCR of different ECOG group in TNBC patients.

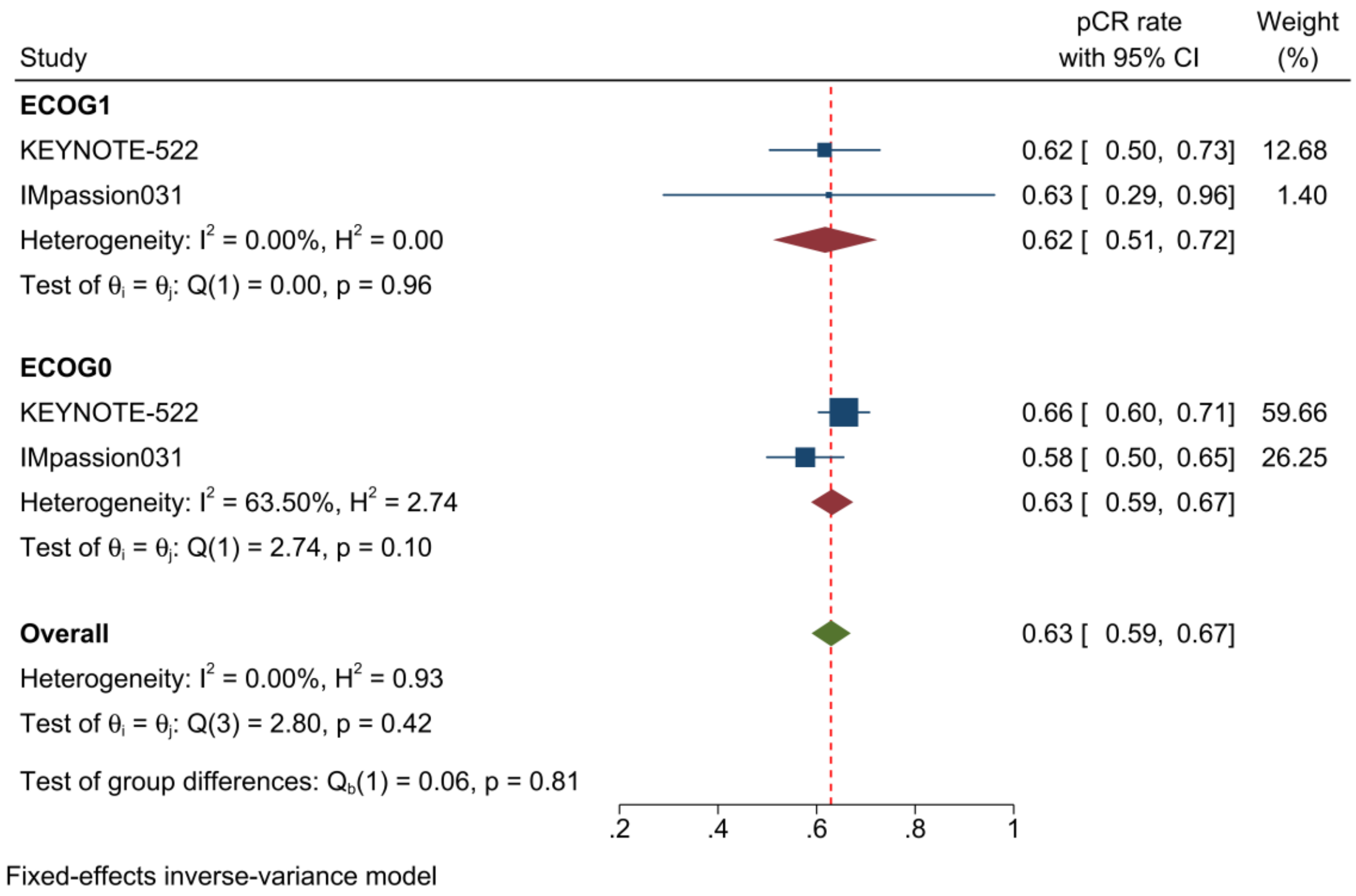

Fig S10 Pooled pCR in TNBC patients treated with PD-1 inhibitors and PD-L1 inhibitors

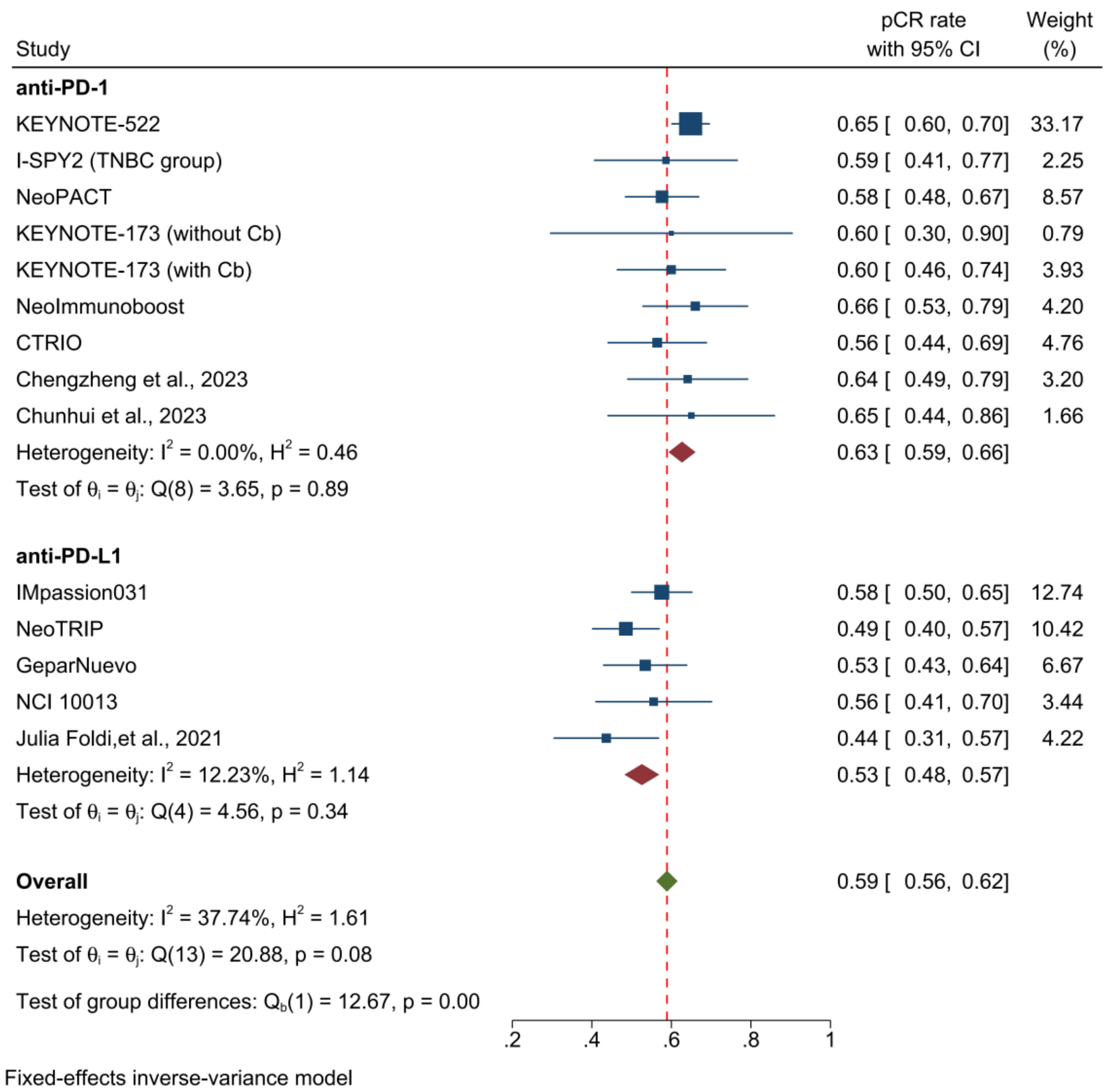

Supplement: Supplementary file 1 [file 12885_2026_16230_MOESM1_ESM.pdf]
